# A cycloaddition product of a chiral maleimide: 4-{(3aS*,6aS*)-4,6-dioxo-1-phenyl-5-[(1*R*)-1-phenyl­ethyl]-1,3a,4,5,6,6a-hexa­hydro­pyrrolo[3,4-*c*]pyrazol-3-yl}phenyl acetate

**DOI:** 10.1107/S1600536809046790

**Published:** 2009-11-14

**Authors:** Chris F. Fronczek, Yaşar Dürüst, Muhammet Yildirim, Frank R. Fronczek

**Affiliations:** a Department of Chemistry, Louisiana State University, Baton Rouge, LA 70803-1804, USA; b Department of Chemistry, Abant Izzet Baysal University, TR-14280, Bolu, Turkey

## Abstract

In the title mol­ecule, C_27_H_23_N_3_O_4_, the two central five-membered rings form a dihedral angle of 63.66 (4)°. The absolute configuration was determined by analysis of Bijvoet pairs based on resonant scattering of light atoms, yielding a Hooft parameter *y* = −0.10 (7).

## Related literature

For cyclo­addition reactions of chiral maleimides with dipolar compounds, see: Bienayme (1997 ▶); Blanarikova *et al.* (2001 ▶); Chihab-Eddine *et al.* (2001 ▶); Oishi *et al.* (1993 ▶, 1999 ▶, 2007 ▶); Ondrus & Fisera (1997 ▶); Tokioka *et al.* (1997 ▶). For the absolute configuration by Bayesian analysis of Bijvoet differences, see: Hooft *et al.* (2008 ▶). For a description of the Cambridge Structural Database, see: Allen (2002 ▶). For related structures, see: Hursthouse *et al.* (2003 ▶); Skof *et al.* (1998 ▶).
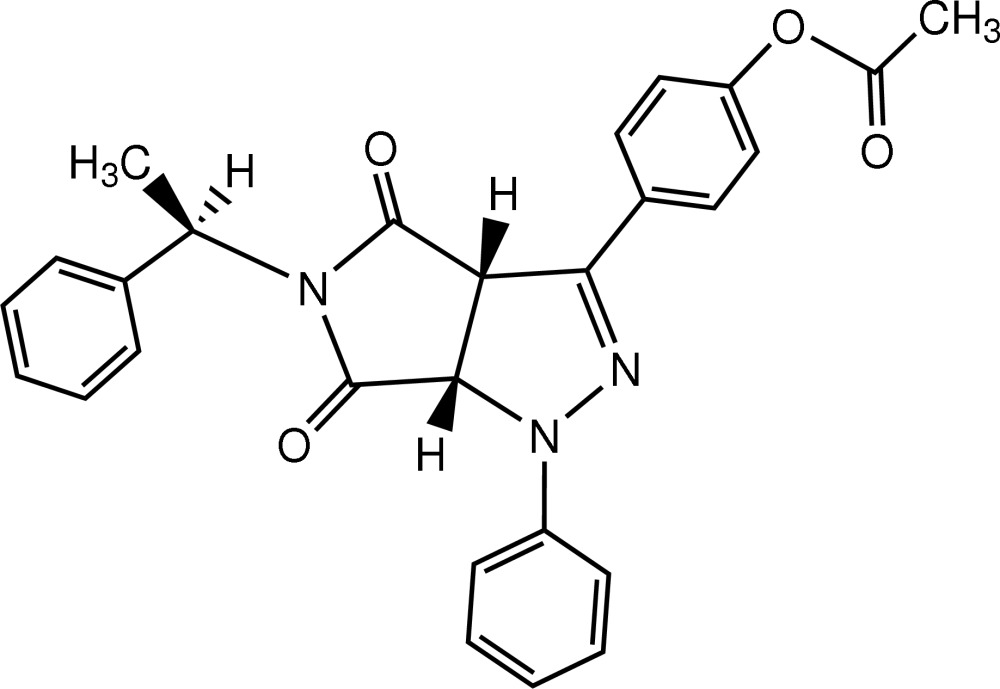


## Experimental

### 

#### Crystal data

C_27_H_23_N_3_O_4_
*M* *_r_* = 453.48
Monoclinic, 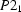

*a* = 9.1391 (5) Å
*b* = 8.7465 (5) Å
*c* = 14.442 (1) Å
β = 103.786 (5)°
*V* = 1121.17 (12) Å^3^
*Z* = 2
Cu *K*α radiation
μ = 0.75 mm^−1^
*T* = 90 K
0.30 × 0.25 × 0.19 mm

#### Data collection

Bruker APEXII CCD diffractometer
Absorption correction: multi-scan (*SADABS*; Sheldrick, 2004 ▶) *T*_min_ = 0.806, *T*_max_ = 0.871
10172 measured reflections
3902 independent reflections
3830 reflections with *I* > 2σ(*I*)
*R*_int_ = 0.029

#### Refinement

*R*[*F* ^2^ > 2σ(*F* ^2^)] = 0.029
*wR*(*F* ^2^) = 0.076
*S* = 1.08
3902 reflections
310 parameters
1 restraint
H-atom parameters constrained
Δρ_max_ = 0.23 e Å^−3^
Δρ_min_ = −0.21 e Å^−3^
Absolute structure: Flack (1983 ▶), 1725 Friedel pairs
Flack parameter: −0.18 (15)

### 

Data collection: *APEX2* (Bruker, 2006 ▶); cell refinement: *SAINT* (Bruker, 2006 ▶); data reduction: *SAINT*; program(s) used to solve structure: *SHELXS97* (Sheldrick, 2008 ▶); program(s) used to refine structure: *SHELXL97* (Sheldrick, 2008 ▶); molecular graphics: *ORTEP-3 for Windows* (Farrugia, 1997 ▶); software used to prepare material for publication: *SHELXTL* (Sheldrick, 2008 ▶).

## Supplementary Material

Crystal structure: contains datablocks global, I. DOI: 10.1107/S1600536809046790/tk2563sup1.cif

Structure factors: contains datablocks I. DOI: 10.1107/S1600536809046790/tk2563Isup2.hkl

Additional supplementary materials:  crystallographic information; 3D view; checkCIF report

## Figures and Tables

**Fig. 1.**
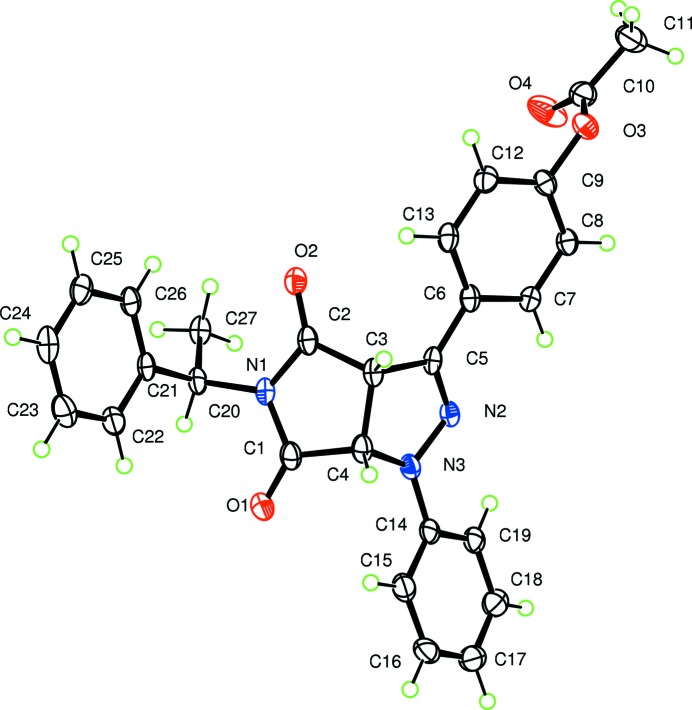
Molecular structure showing atom labelling and displacement ellipsoids at the 50% level, with H atoms having arbitrary radius.

**Fig. 2.**
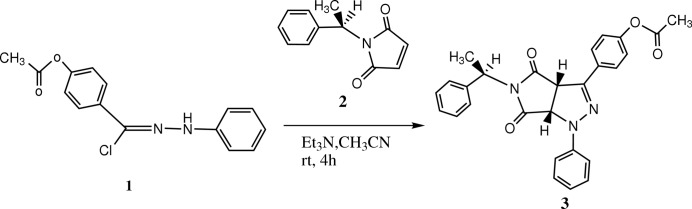
The formation of the title compound.

## supplementary crystallographic information

### Comment

There are limited examples of cycloaddition reactions of chiral maleimides with dipolar compounds like nitrones, nitriloxides and anthrones reported in the literature (Bienayme, 1997; Blanarikova *et al.*, 2001; Chihab-Eddine *et al.*, 2001; Oishi *et al.*, 1993; 1999; 2007; Ondrus & Fisera, 1997; Tokioka *et al.*, 1997). To our best knowledge, a literature search revealed that 1,3-dipolar cycloaddition of C,*N*-substituted nitrilimines to the chiral maleimide, (*R*)—*N*-(1-phenylethyl) maleimide, has not been studied. In this work, we report the synthesis, characterization and crystal structure of the diastereomer obtained from the above reaction.

The two five-membered rings at the core of this molecule form a dihedral angle of 63.66 (4)°, and the two rings themselves are essentially planar. The mean deviation of the seven pyrrolidine-2,5-dione atoms from their least-squares plane is 0.008 Å, and the mean deviation for the 4,5-dihydro-1*H*-pyrazole ring is 0.021 Å. Atom N1 in the pyrrolidine-2,5-dione deviates most from the plane, 0.0172 (11) Å. Atom C4 deviates most from the 4,5-dihydro-1*H*-pyrazole ring, with deviation 0.0315 (9) Å. Only two structures with similar cores are found in the Cambridge Database (Allen, 2002, version 5.30, Nov. 2008), refcodes CIRFEP (Hursthouse *et al.*, 2003) and WIQBIH (Skof *et al.*, 1998). In CIRFEP, the dihedral angle between the central ring planes is 63.65 (9)°, for one of two independent molecules and 64.23 (9)° for the other. For WIQBIH, the dihedral angle formed by the central ring planes 65.99 (6)°.

In the title compound, the acetate group is nearly orthogonal to the phenyl ring to which it is bonded, as shown by the torsion angle C10—O3—C9—C8, 80.13 (17)°. The phenyl group containing C6 is rotated out of the 4,5-dihydro-1*H*-pyrazole plane with a torsion angle (C3—C5—C6—C7) of 167.48 (13)°. In addition, the phenyl group containing atom C14 is rotated out of the same plane with a torsion angle (N2—N3—C14—C15) of 159.64 (15)°.

The absolute configuration was determined by refinement of the Flack (1983) parameter, based on resonant scattering of the light atoms. The assignment agrees with that of the starting materials. Analysis of the Bijvoet pairs using the method of Hooft *et al.* (2008) yielded *y* = -0.10 (7) for this structure, confirming the absolute configuration.

### Experimental

*C*-(4-Acetoxyphenyl)-*N*-phenyl hydrazonyl chloride *1* (0,144 g,0.5 mmol) and (*R*)—*N*-(1-phenylethyl) maleimide *2* (0,100 g, 0.5 mmol) were dissolved in dry acetonitrile (20 ml). Et_3_N (0.404 g, 4 mmol) was added dropwise into the mixture with stirring and after the addition was completed, the reaction mixture was stirred at room temperature for 2 h; the progress of the reaction was monitored by TLC. The acetonitrile was evaporated under reduced pressure and the reaction mixture was taken into water (50 ml) to remove Et_3_N.HCl. The crude brown cycloadduct that precipitated was filtered and washed thoroughly with water and then hexanes, and dried under vacuum. After purification on Chromatotron (Centrifugal Thin-Layer Chromatograph) using n-hexane-ethyl acetate (2:1) as eluant and recrystallization from a mixture of dichloromethane-n-hexane-acetone, the cycloadduct *3* was isolated as yellow needles (160 mg, 71%). [α]^21°C^_589_ = +79.0° (c = 0.01 g/ml, l=10 cm, acetone). M. pt. 359–361 K. *R*_f_: 0.60 (ethyl acetate-n-hexane; 1:2).

IR (KBr): ν = 1757 (CH_3_CO), 1710 (CO), 1599 (CN), 1498, 1452, 1357, 1197, 750 cm^-1^.

^1^H NMR (400 MHz, CDCl_3_): δ = 8.10 (q, J=3.8 Hz, 2H), 7.58 (t, J=7.6 Hz, 2H), 7.47 (t, J=7.2 Hz, 2H), 7.24–7.36 (m, 5H), 7.18 (q, J=4.6 Hz, 2H), 7.01 (t, J=7.3 Hz, 1H), 5.44 (quintet, 1H, CH_3_CH), 5.08–4.95 (dd, J=51.1 10.9 Hz, 1H,), 4.76 (dd, J=21.5 11.0 Hz, 1H), 2.35 (s, 3H) 1.76–1.86 (m, 3H).

^13^C NMR (100 MHz,CDCl_3_): δ =172.5 (CO), 171.5 (CO), 169.4 (CH_3_CO), 151.5 (CN), 144.5, 142.0, 138.7, 129.2, 128.9, 128.6, 128.3, 128.2, 127.6, 121.8, 121.5, 114.4, 65.4 (–CH), 53.3 (–CH), 51.4 (–CH), 21.1(CH_3_CO), 16.4 (CH_3_).

GC—MS (70 eV): (m/*z*, %)= 453 (100) [*M*]^+^, 411 (65), 307 (100), 236 (50), 207 (10), 105 (33), 70 (10).

Anal Calcd for C_27_H_23_N_3_O_4_. C, 71.51; H, 5.11; N, 9.27%; found C, 71.63; H, 5.11; N, 9.25%.

### Refinement

H atoms on C were placed in idealized positions with C—H distances 0.95 - 1.00 Å and thereafter treated as riding. A torsional parameter was refined for each methyl group. *U*_iso_ for H were assigned as 1.2 times *U*_eq_ of the attached atoms (1.5 for methyl).

### Crystal data

**Table:**

C_27_H_23_N_3_O_4_	*F*(000) = 476
*M**_r_* = 453.48	*D*_x_ = 1.343 Mg m^−^^3^
Monoclinic, *P*2_1_	Cu *K*α radiation, λ = 1.54178 Å
Hall symbol: P 2yb	Cell parameters from 7860 reflections
*a* = 9.1391 (5) Å	θ = 3.2–68.3°
*b* = 8.7465 (5) Å	µ = 0.75 mm^−^^1^
*c* = 14.442 (1) Å	*T* = 90 K
β = 103.786 (5)°	Fragment, yellow
*V* = 1121.17 (12) Å^3^	0.30 × 0.25 × 0.19 mm
*Z* = 2	

### Data collection

**Table:**

Bruker APEXII CCD diffractometer	3902 independent reflections
Radiation source: fine-focus sealed tube	3830 reflections with *I* > 2σ(*I*)
graphite	*R*_int_ = 0.029
φ and ω scans	θ_max_ = 68.8°, θ_min_ = 3.2°
Absorption correction: multi-scan (*SADABS*; Sheldrick, 2004)	*h* = −10→11
*T*_min_ = 0.806, *T*_max_ = 0.871	*k* = −9→10
10172 measured reflections	*l* = −17→17

### Refinement

**Table:**

Refinement on *F*^2^	Hydrogen site location: inferred from neighbouring sites
Least-squares matrix: full	H-atom parameters constrained
*R*[*F*^2^ > 2σ(*F*^2^)] = 0.029	*w* = 1/[σ^2^(*F*_o_^2^) + (0.0355*P*)^2^ + 0.2144*P*] where *P* = (*F*_o_^2^ + 2*F*_c_^2^)/3
*wR*(*F*^2^) = 0.076	(Δ/σ)_max_ = 0.001
*S* = 1.08	Δρ_max_ = 0.23 e Å^−^^3^
3902 reflections	Δρ_min_ = −0.21 e Å^−^^3^
310 parameters	Extinction correction: *SHELXL97* (Sheldrick, 2008), Fc^*^=kFc[1+0.001xFc^2^λ^3^/sin(2θ)]^-1/4^
1 restraint	Extinction coefficient: 0.0021 (4)
Primary atom site location: structure-invariant direct methods	Absolute structure: Flack (1983), 1725 Friedel pairs
Secondary atom site location: difference Fourier map	Flack parameter: −0.18 (15)

### Special details

**Table:**

Geometry. All e.s.d.'s (except the e.s.d. in the dihedral angle between two l.s. planes) are estimated using the full covariance matrix. The cell e.s.d.'s are taken into account individually in the estimation of e.s.d.'s in distances, anglesand torsion angles; correlations between e.s.d.'s in cell parameters are only used when they are defined by crystal symmetry. An approximate (isotropic) treatment of cell e.s.d.'s is used for estimating e.s.d.'s involving l.s. planes.
Refinement. Refinement of *F*^2^ against ALL reflections. The weighted *R*-factor *wR* and goodness of fit *S* are based on *F*^2^, conventional *R*-factors *R* are based on *F*, with *F* set to zero for negative *F*^2^. The threshold expression of *F*^2^ > σ(*F*^2^) is used only for calculating *R*-factors(gt) *etc*. and is not relevant to the choice of reflections for refinement. *R*-factors based on *F*^2^ are statistically about twice as large as those based on *F*, and *R*- factors based on ALL data will be even larger.

### Fractional atomic coordinates and isotropic or equivalent isotropic displacement parameters (Å^2^)

**Table:**

	*x*	*y*	*z*	*U*_iso_*/*U*_eq_	
O1	0.31157 (12)	0.46611 (15)	0.83990 (8)	0.0332 (3)	
O2	0.25737 (11)	0.33703 (12)	0.52738 (7)	0.0246 (2)	
O3	−0.37802 (12)	0.37334 (12)	0.19079 (8)	0.0270 (2)	
O4	−0.29475 (18)	0.60155 (17)	0.15371 (9)	0.0512 (4)	
N1	0.31609 (13)	0.41517 (15)	0.68415 (9)	0.0217 (3)	
N2	−0.10053 (12)	0.50276 (15)	0.63294 (8)	0.0202 (3)	
N3	−0.01685 (13)	0.48013 (15)	0.72440 (9)	0.0223 (3)	
C1	0.25163 (16)	0.41734 (19)	0.76244 (11)	0.0243 (3)	
C2	0.22410 (15)	0.35331 (16)	0.60278 (11)	0.0214 (3)	
C3	0.07344 (15)	0.31006 (17)	0.62509 (10)	0.0212 (3)	
H3	0.0497	0.1990	0.6138	0.025*	
C4	0.09114 (16)	0.35455 (18)	0.72977 (11)	0.0238 (3)	
H4	0.0712	0.2675	0.7699	0.029*	
C5	−0.05658 (15)	0.41174 (17)	0.57491 (10)	0.0200 (3)	
C6	−0.13497 (15)	0.40425 (17)	0.47391 (10)	0.0202 (3)	
C7	−0.27083 (15)	0.48411 (17)	0.44207 (10)	0.0201 (3)	
H7	−0.3091	0.5440	0.4859	0.024*	
C8	−0.34965 (16)	0.47673 (18)	0.34774 (10)	0.0220 (3)	
H8	−0.4417	0.5309	0.3265	0.026*	
C9	−0.29238 (16)	0.38923 (18)	0.28474 (10)	0.0229 (3)	
C10	−0.37251 (19)	0.4912 (2)	0.13033 (11)	0.0306 (4)	
C11	−0.4770 (2)	0.4640 (2)	0.03545 (12)	0.0389 (4)	
H11A	−0.5801	0.4895	0.0386	0.058*	
H11B	−0.4722	0.3562	0.0178	0.058*	
H11C	−0.4473	0.5285	−0.0125	0.058*	
C12	−0.15893 (16)	0.31088 (18)	0.31350 (11)	0.0256 (3)	
H12	−0.1211	0.2523	0.2689	0.031*	
C13	−0.07975 (16)	0.31808 (17)	0.40842 (11)	0.0235 (3)	
H13	0.0125	0.2640	0.4288	0.028*	
C14	−0.06694 (16)	0.54178 (18)	0.80081 (10)	0.0227 (3)	
C15	−0.01474 (17)	0.4835 (2)	0.89311 (11)	0.0295 (3)	
H15	0.0588	0.4046	0.9053	0.035*	
C16	−0.07195 (18)	0.5425 (2)	0.96665 (11)	0.0336 (4)	
H16	−0.0387	0.5013	1.0290	0.040*	
C17	−0.17628 (19)	0.6599 (2)	0.95098 (12)	0.0337 (4)	
H17	−0.2148	0.6987	1.0019	0.040*	
C18	−0.22385 (19)	0.7203 (2)	0.85994 (12)	0.0309 (4)	
H18	−0.2947	0.8017	0.8486	0.037*	
C19	−0.16884 (16)	0.66269 (18)	0.78508 (11)	0.0241 (3)	
H19	−0.2008	0.7059	0.7232	0.029*	
C20	0.47221 (15)	0.47134 (18)	0.69157 (10)	0.0224 (3)	
H20	0.5005	0.5331	0.7515	0.027*	
C21	0.58097 (15)	0.33623 (17)	0.70440 (10)	0.0216 (3)	
C22	0.64427 (16)	0.2851 (2)	0.79644 (11)	0.0280 (3)	
H22	0.6183	0.3338	0.8491	0.034*	
C23	0.74501 (18)	0.1637 (2)	0.81217 (12)	0.0336 (4)	
H23	0.7873	0.1297	0.8753	0.040*	
C24	0.78404 (17)	0.09222 (19)	0.73593 (13)	0.0306 (4)	
H24	0.8537	0.0098	0.7467	0.037*	
C25	0.72090 (17)	0.14149 (18)	0.64378 (12)	0.0262 (3)	
H25	0.7467	0.0923	0.5912	0.031*	
C26	0.61966 (15)	0.26322 (18)	0.62843 (10)	0.0231 (3)	
H26	0.5767	0.2966	0.5652	0.028*	
C27	0.47789 (16)	0.57928 (18)	0.60983 (11)	0.0248 (3)	
H27A	0.4575	0.5219	0.5498	0.037*	
H27B	0.5780	0.6260	0.6211	0.037*	
H27C	0.4017	0.6595	0.6059	0.037*	

### Atomic displacement parameters (Å^2^)

**Table:**

	*U*^11^	*U*^22^	*U*^33^	*U*^12^	*U*^13^	*U*^23^
O1	0.0219 (5)	0.0485 (8)	0.0267 (6)	−0.0021 (5)	0.0010 (4)	0.0012 (5)
O2	0.0188 (5)	0.0242 (6)	0.0303 (6)	−0.0012 (4)	0.0046 (4)	−0.0025 (4)
O3	0.0277 (5)	0.0241 (6)	0.0265 (5)	−0.0044 (4)	0.0012 (4)	−0.0038 (4)
O4	0.0695 (10)	0.0493 (9)	0.0298 (6)	−0.0348 (8)	0.0020 (6)	0.0011 (6)
N1	0.0143 (5)	0.0213 (6)	0.0282 (6)	0.0002 (5)	0.0022 (5)	0.0005 (5)
N2	0.0155 (5)	0.0196 (6)	0.0237 (6)	−0.0022 (5)	0.0013 (5)	0.0033 (5)
N3	0.0171 (6)	0.0252 (6)	0.0227 (6)	0.0016 (5)	0.0007 (4)	0.0034 (5)
C1	0.0183 (7)	0.0242 (8)	0.0282 (8)	0.0028 (6)	0.0015 (6)	0.0066 (6)
C2	0.0161 (6)	0.0147 (7)	0.0311 (8)	0.0021 (6)	0.0011 (6)	0.0024 (6)
C3	0.0156 (7)	0.0165 (7)	0.0298 (7)	−0.0005 (5)	0.0020 (5)	0.0027 (6)
C4	0.0170 (7)	0.0233 (8)	0.0295 (7)	0.0021 (6)	0.0025 (6)	0.0057 (6)
C5	0.0134 (6)	0.0155 (7)	0.0308 (8)	−0.0005 (5)	0.0046 (5)	0.0022 (6)
C6	0.0159 (6)	0.0147 (7)	0.0288 (7)	−0.0021 (5)	0.0028 (5)	0.0017 (6)
C7	0.0181 (6)	0.0164 (7)	0.0265 (7)	−0.0001 (6)	0.0063 (5)	−0.0004 (6)
C8	0.0189 (7)	0.0180 (7)	0.0274 (7)	0.0024 (6)	0.0024 (6)	0.0015 (6)
C9	0.0221 (7)	0.0197 (8)	0.0249 (7)	−0.0031 (6)	0.0013 (6)	−0.0013 (6)
C10	0.0314 (8)	0.0342 (10)	0.0267 (8)	−0.0062 (7)	0.0079 (6)	−0.0026 (7)
C11	0.0424 (10)	0.0454 (11)	0.0263 (8)	−0.0104 (9)	0.0031 (7)	−0.0024 (8)
C12	0.0226 (7)	0.0221 (8)	0.0325 (8)	0.0001 (6)	0.0073 (6)	−0.0063 (6)
C13	0.0165 (7)	0.0185 (7)	0.0341 (8)	0.0016 (5)	0.0035 (6)	−0.0011 (6)
C14	0.0167 (7)	0.0264 (8)	0.0239 (7)	−0.0085 (6)	0.0029 (6)	−0.0010 (6)
C15	0.0219 (7)	0.0358 (9)	0.0285 (8)	−0.0018 (7)	0.0015 (6)	0.0056 (7)
C16	0.0289 (8)	0.0481 (11)	0.0222 (7)	−0.0142 (8)	0.0027 (6)	0.0000 (7)
C17	0.0307 (8)	0.0419 (10)	0.0304 (8)	−0.0143 (8)	0.0113 (7)	−0.0105 (7)
C18	0.0287 (8)	0.0302 (9)	0.0350 (9)	−0.0049 (7)	0.0098 (7)	−0.0072 (7)
C19	0.0194 (7)	0.0246 (8)	0.0274 (8)	−0.0051 (6)	0.0034 (6)	−0.0025 (6)
C20	0.0137 (6)	0.0224 (7)	0.0289 (7)	−0.0026 (6)	0.0010 (5)	−0.0025 (6)
C21	0.0120 (6)	0.0211 (8)	0.0298 (7)	−0.0046 (5)	0.0010 (5)	0.0008 (6)
C22	0.0207 (7)	0.0316 (9)	0.0292 (8)	−0.0005 (7)	0.0010 (6)	−0.0008 (7)
C23	0.0256 (8)	0.0361 (10)	0.0342 (9)	0.0025 (7)	−0.0026 (7)	0.0074 (7)
C24	0.0179 (7)	0.0210 (8)	0.0495 (10)	0.0000 (6)	0.0011 (7)	0.0053 (7)
C25	0.0187 (7)	0.0220 (8)	0.0382 (9)	−0.0049 (6)	0.0073 (6)	−0.0028 (6)
C26	0.0160 (7)	0.0213 (8)	0.0302 (7)	−0.0049 (6)	0.0022 (5)	0.0031 (6)
C27	0.0176 (7)	0.0203 (7)	0.0354 (8)	−0.0002 (6)	0.0043 (6)	0.0019 (6)

### Geometric parameters (Å, °)

**Table:**

O1—C1	1.201 (2)	C12—H12	0.9500
O2—C2	1.2068 (18)	C13—H13	0.9500
O3—C10	1.359 (2)	C14—C19	1.392 (2)
O3—C9	1.4024 (17)	C14—C15	1.401 (2)
O4—C10	1.199 (2)	C15—C16	1.390 (2)
N1—C2	1.3818 (19)	C15—H15	0.9500
N1—C1	1.3940 (19)	C16—C17	1.383 (3)
N1—C20	1.4886 (17)	C16—H16	0.9500
N2—C5	1.288 (2)	C17—C18	1.387 (3)
N2—N3	1.3739 (16)	C17—H17	0.9500
N3—C14	1.3995 (19)	C18—C19	1.391 (2)
N3—C4	1.4663 (19)	C18—H18	0.9500
C1—C4	1.532 (2)	C19—H19	0.9500
C2—C3	1.5343 (19)	C20—C27	1.522 (2)
C3—C5	1.5224 (19)	C20—C21	1.527 (2)
C3—C4	1.532 (2)	C20—H20	1.0000
C3—H3	1.0000	C21—C26	1.386 (2)
C4—H4	1.0000	C21—C22	1.391 (2)
C5—C6	1.465 (2)	C22—C23	1.388 (2)
C6—C13	1.395 (2)	C22—H22	0.9500
C6—C7	1.403 (2)	C23—C24	1.385 (3)
C7—C8	1.382 (2)	C23—H23	0.9500
C7—H7	0.9500	C24—C25	1.387 (2)
C8—C9	1.384 (2)	C24—H24	0.9500
C8—H8	0.9500	C25—C26	1.393 (2)
C9—C12	1.374 (2)	C25—H25	0.9500
C10—C11	1.491 (2)	C26—H26	0.9500
C11—H11A	0.9800	C27—H27A	0.9800
C11—H11B	0.9800	C27—H27B	0.9800
C11—H11C	0.9800	C27—H27C	0.9800
C12—C13	1.391 (2)		
			
C10—O3—C9	116.66 (12)	C13—C12—H12	120.3
C2—N1—C1	113.95 (12)	C12—C13—C6	120.38 (13)
C2—N1—C20	124.68 (12)	C12—C13—H13	119.8
C1—N1—C20	121.34 (12)	C6—C13—H13	119.8
C5—N2—N3	110.38 (12)	C19—C14—N3	119.74 (13)
N2—N3—C14	119.40 (12)	C19—C14—C15	119.67 (14)
N2—N3—C4	111.85 (12)	N3—C14—C15	120.60 (14)
C14—N3—C4	126.12 (12)	C16—C15—C14	119.16 (15)
O1—C1—N1	124.99 (14)	C16—C15—H15	120.4
O1—C1—C4	127.24 (14)	C14—C15—H15	120.4
N1—C1—C4	107.70 (12)	C17—C16—C15	121.39 (16)
O2—C2—N1	125.48 (12)	C17—C16—H16	119.3
O2—C2—C3	126.31 (13)	C15—C16—H16	119.3
N1—C2—C3	108.21 (12)	C16—C17—C18	119.08 (16)
C5—C3—C4	102.02 (12)	C16—C17—H17	120.5
C5—C3—C2	113.18 (12)	C18—C17—H17	120.5
C4—C3—C2	104.81 (11)	C17—C18—C19	120.61 (17)
C5—C3—H3	112.1	C17—C18—H18	119.7
C4—C3—H3	112.1	C19—C18—H18	119.7
C2—C3—H3	112.1	C18—C19—C14	120.01 (15)
N3—C4—C1	109.32 (13)	C18—C19—H19	120.0
N3—C4—C3	103.10 (11)	C14—C19—H19	120.0
C1—C4—C3	105.26 (11)	N1—C20—C27	110.98 (12)
N3—C4—H4	112.8	N1—C20—C21	109.78 (12)
C1—C4—H4	112.8	C27—C20—C21	115.64 (12)
C3—C4—H4	112.8	N1—C20—H20	106.6
N2—C5—C6	121.41 (13)	C27—C20—H20	106.6
N2—C5—C3	112.37 (12)	C21—C20—H20	106.6
C6—C5—C3	126.07 (13)	C26—C21—C22	118.81 (14)
C13—C6—C7	118.79 (13)	C26—C21—C20	122.82 (13)
C13—C6—C5	121.95 (13)	C22—C21—C20	118.37 (13)
C7—C6—C5	119.25 (13)	C23—C22—C21	120.72 (15)
C8—C7—C6	120.82 (14)	C23—C22—H22	119.6
C8—C7—H7	119.6	C21—C22—H22	119.6
C6—C7—H7	119.6	C24—C23—C22	120.12 (15)
C7—C8—C9	118.94 (13)	C24—C23—H23	119.9
C7—C8—H8	120.5	C22—C23—H23	119.9
C9—C8—H8	120.5	C23—C24—C25	119.70 (15)
C12—C9—C8	121.64 (14)	C23—C24—H24	120.2
C12—C9—O3	119.57 (13)	C25—C24—H24	120.2
C8—C9—O3	118.67 (13)	C24—C25—C26	119.92 (15)
O4—C10—O3	122.68 (15)	C24—C25—H25	120.0
O4—C10—C11	126.45 (17)	C26—C25—H25	120.0
O3—C10—C11	110.86 (15)	C21—C26—C25	120.73 (14)
C10—C11—H11A	109.5	C21—C26—H26	119.6
C10—C11—H11B	109.5	C25—C26—H26	119.6
H11A—C11—H11B	109.5	C20—C27—H27A	109.5
C10—C11—H11C	109.5	C20—C27—H27B	109.5
H11A—C11—H11C	109.5	H27A—C27—H27B	109.5
H11B—C11—H11C	109.5	C20—C27—H27C	109.5
C9—C12—C13	119.42 (14)	H27A—C27—H27C	109.5
C9—C12—H12	120.3	H27B—C27—H27C	109.5
			
C5—N2—N3—C14	−165.83 (13)	C7—C8—C9—C12	−0.5 (2)
C5—N2—N3—C4	−3.11 (16)	C7—C8—C9—O3	175.50 (13)
C2—N1—C1—O1	−179.89 (16)	C10—O3—C9—C12	−103.74 (17)
C20—N1—C1—O1	1.9 (2)	C10—O3—C9—C8	80.13 (17)
C2—N1—C1—C4	−2.61 (17)	C9—O3—C10—O4	2.7 (2)
C20—N1—C1—C4	179.13 (13)	C9—O3—C10—C11	−175.85 (14)
C1—N1—C2—O2	−178.43 (14)	C8—C9—C12—C13	0.7 (2)
C20—N1—C2—O2	−0.2 (2)	O3—C9—C12—C13	−175.35 (13)
C1—N1—C2—C3	1.78 (16)	C9—C12—C13—C6	−0.1 (2)
C20—N1—C2—C3	179.98 (13)	C7—C6—C13—C12	−0.5 (2)
O2—C2—C3—C5	−69.64 (19)	C5—C6—C13—C12	178.55 (14)
N1—C2—C3—C5	110.15 (14)	N2—N3—C14—C19	−20.74 (19)
O2—C2—C3—C4	−179.98 (14)	C4—N3—C14—C19	179.21 (13)
N1—C2—C3—C4	−0.19 (15)	N2—N3—C14—C15	159.64 (14)
N2—N3—C4—C1	116.74 (13)	C4—N3—C14—C15	−0.4 (2)
C14—N3—C4—C1	−81.94 (17)	C19—C14—C15—C16	3.3 (2)
N2—N3—C4—C3	5.15 (15)	N3—C14—C15—C16	−177.05 (14)
C14—N3—C4—C3	166.47 (13)	C14—C15—C16—C17	−1.6 (2)
O1—C1—C4—N3	69.3 (2)	C15—C16—C17—C18	−0.4 (2)
N1—C1—C4—N3	−107.87 (14)	C16—C17—C18—C19	0.7 (2)
O1—C1—C4—C3	179.49 (16)	C17—C18—C19—C14	1.0 (2)
N1—C1—C4—C3	2.28 (16)	N3—C14—C19—C18	177.33 (14)
C5—C3—C4—N3	−4.89 (14)	C15—C14—C19—C18	−3.1 (2)
C2—C3—C4—N3	113.31 (12)	C2—N1—C20—C27	49.95 (19)
C5—C3—C4—C1	−119.45 (12)	C1—N1—C20—C27	−131.99 (14)
C2—C3—C4—C1	−1.24 (15)	C2—N1—C20—C21	−79.14 (17)
N3—N2—C5—C6	175.25 (12)	C1—N1—C20—C21	98.93 (15)
N3—N2—C5—C3	−0.51 (16)	N1—C20—C21—C26	92.06 (16)
C4—C3—C5—N2	3.60 (15)	C27—C20—C21—C26	−34.43 (19)
C2—C3—C5—N2	−108.46 (14)	N1—C20—C21—C22	−88.28 (16)
C4—C3—C5—C6	−171.92 (13)	C27—C20—C21—C22	145.23 (14)
C2—C3—C5—C6	76.02 (18)	C26—C21—C22—C23	0.3 (2)
N2—C5—C6—C13	173.31 (14)	C20—C21—C22—C23	−179.33 (14)
C3—C5—C6—C13	−11.5 (2)	C21—C22—C23—C24	0.2 (2)
N2—C5—C6—C7	−7.7 (2)	C22—C23—C24—C25	−0.6 (2)
C3—C5—C6—C7	167.48 (13)	C23—C24—C25—C26	0.5 (2)
C13—C6—C7—C8	0.6 (2)	C22—C21—C26—C25	−0.4 (2)
C5—C6—C7—C8	−178.46 (13)	C20—C21—C26—C25	179.21 (13)
C6—C7—C8—C9	−0.1 (2)	C24—C25—C26—C21	0.0 (2)
